# Nanofabrication of High-Resolution Periodic Structures with a Gap Size Below 100 nm by Two-Photon Polymerization

**DOI:** 10.1186/s11671-019-2955-5

**Published:** 2019-04-17

**Authors:** Lei Zheng, Kestutis Kurselis, Ayman El-Tamer, Ulf Hinze, Carsten Reinhardt, Ludger Overmeyer, Boris Chichkov

**Affiliations:** 1 Laboratory of Nano and Quantum Engineering, Leibniz Universität Hannover, Schneiderberg 39, Hannover, 30167 Germany; 2 Laser Zentrum Hannover e.V., Hollerithallee 8, Hannover, 30419 Germany; 3 Institute of Quantum Optics, Leibniz Universität Hannover, Welfengarten 1, Hannover, 30167 Germany; 4 Institute of Transport and Automation Technology, Leibniz Universität Hannover, An der Universität 2, Garbsen, 30823 Germany; 5 Hochschule Bremen, Neustadtswall 30, Bremen, 28199 Germany

**Keywords:** Nanofabrication, Two-photon polymerization, Sub-100 nm, Periodic structures

## Abstract

In this paper, approaches for the realization of high-resolution periodic structures with gap sizes at sub-100 nm scale by two-photon polymerization (2PP) are presented. The impact of laser intensity on the feature sizes and surface quality is investigated. The influence of different photosensitive materials on the structure formation is compared. Based on the elliptical geometry character of the voxel, the authors present an idea to realize high-resolution structures with feature sizes less than 100 nm by controlling the laser focus position with respect to the glass substrate. This investigation covers structures fabricated respectively in the plane along and perpendicular to the major axis of voxel. The authors also provide a useful approach to manage the fabrication of proposed periodic structure with a periodic distance of 200 nm and a gap size of 65 nm.

## Introduction

The demand for the downscaling of devices grows rapidly with the continuous progress of nanotechnology in recent years. The miniaturized structures with feature sizes below the diffraction limit can be applied in various fields like plasmonics [1], micro- and nanooptics [2], nanophotonics [3, 4], and biomedicine [5, 6]. Moreover, structures with sub-wavelength dimensions are also able to facilitate the characterization performance at micro- and nanoscale [7, 8]. For example, tips [9] and nanoanttennas [10] can be used to improve the characterization performance of high-resolution structures by enhancing the light confinement in the near-field, and gratings [11] are able to transform optical information from the near field to the far field.

As to the realization of high-resolution structures, two-photon polymerization (2PP) is popularly utilized due to its capabilities of achieving high resolution and 3D fabrication [12]. Two-photon polymerization is a manufacturing method based on two-photon absorption (2PA), which is a nonlinear process that theoretically enables the achievement of resolution below the diffraction limit. Various 2PP-based methods, such as adding photoinitiator with a high initiation efficiency [13], shaping the spatial phase of deactivation beam [14], using sub-10 fs [15] and 520-nm femtosecond laser pulses [16], combining with hybrid optics [17] and a developed sub-diffraction optical beam lithography [18], have been applied to realize feature sizes at sub-100 nm scale. However, these sizes are mostly achieved on suspended lines or a single line. It still remains challenging to experimentally realize feature sizes and gap sizes beyond the diffraction limit in periodic structures due to the radical diffusion exchanging effect in the gap region when center-to-center distance between adjacent features gets very close [19]. Nevertheless, a few strategies were demonstrated for the purpose of achieving periodic structures with a nanoscale gap distance. Photonic crystals with a periodic distance of 400 nm were realized by adding a quencher molecule into the photoresist [20]. With this approach, the gap size between adjacent lines of the photonic crystals is around 300 nm. Moreover, grating lines with a periodic distance of 175 nm and a gap size of 75 nm were achieved by a STED lithography technique [19]. Recently, it was presented that a straight forward thermal post-treatment process of samples by calcination is able to realize feature sizes down to approximately 85 nm [21]. The above approaches have afforded for the realization of periodic structures with gap sizes below the diffraction limit. However, they are quite special with higher cost, more complicated operations and procedures comparing to 2PP.

**Fig. 1:**
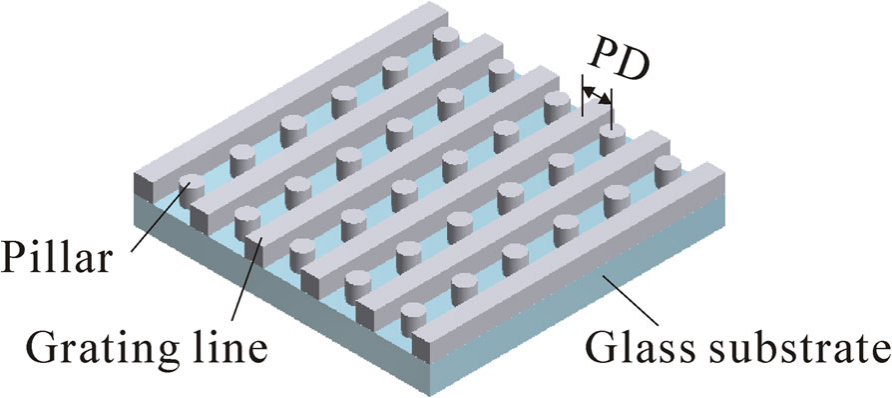
Schematic illustration of the proposed periodic structure. The periodic distance between adjacent features is represented by *PD*

In this paper, an experimental investigation on the realization of a periodic device (Fig. 1) with both feature sizes and gap sizes below the diffraction limit using 2PP is carried out. The high-resolution periodic structure, composed of grating lines with pillars periodically located between them, was proposed for the enhancement of characterization resolution of interferometric Fourier transform scatterometry (IFTS) [22, 23], which is a method for the characterization of micro- and nanostructures. It is known that the spatial resolution of structures is mainly determined by the photosensitive materials, optical system, and processing parameters [15]. Specifically, researchers have reported that the orientation of laser beam polarization can affect the structure dimensions [24]. When a laser is linearly polarized parallel to its scanning direction, a minimum feature dimension can be realized. Therefore, the laser employed in the experiments is equipped with a linear polarization parallel to the laser scanning direction for the purpose of obtaining smaller feature sizes. Based on this configuration, the effect of laser intensity on the feature sizes is investigated first. Then, the influence of different photosensitive materials on the structure formation is compared. When laser directly writes structures on a glass substrate, only part of the voxel polymerizes the photoresist because the other part of the voxel is inside glass substrate. Benefiting from the elliptical geometry of voxel, an idea of reducing the feature size and gap size by controlling laser focus position with respect to the glass substrate is specially presented. The feature sizes of grating lines (fabricated in the plane perpendicular to the major axis of voxel) and pillars (fabricated in the plane along the major axis of voxel) depending on relative laser focus positions are respectively investigated. As a result, grating lines with a minimum width of 78 nm and pillars with the diameter of 110 nm are realized. In addition, the proposed structure with an area size of 20×20 *μ*m, a periodic distance of 200 nm, and a gap size of 65 nm is demonstrated by separately fabricating grating lines and pillars.

## Methods

### Fabrication Method

**Fig. 2:**
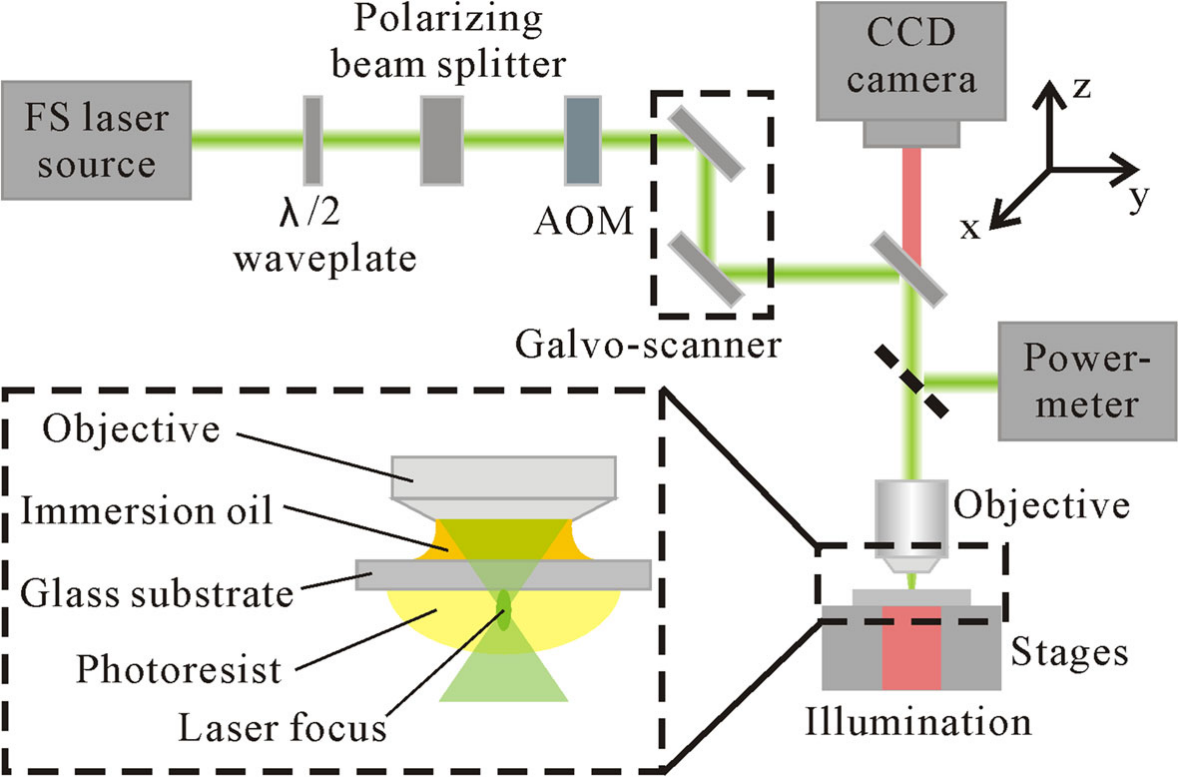
Schematic diagram of 2PP fabrication system

The structures presented in this paper were fabricated using two-photon polymerization. A schematic illustration of the experimental setup is shown in Fig. 2. This 2PP fabrication system, which is also available commercially [25, 26], is able to coordinate all axis simultaneously and reach the velocity over the full travel range without stepping and stitching at a speed of up to 50 mm/s. A linear polarized femtosecond laser with a frequency doubled output at 513 nm, a pulse width of 60 fs and a repetition rate of 76 MHz is used. Laser power is controlled by a half wave plate and a polarizing beam splitter cube. Highly accurate air-bearing translation stages with a travel range of 15 cm are employed as well. A CCD camera is mounted for online monitoring. The polymerization process can be monitored by a CCD camera due to the refractive index variation of photoresist induced by the polymerization. The sample consists of a droplet of photosensitive material on the glass substrate, which is fixed to the translation stage with photoresist on the bottom side. Laser beam is focused into the photoresist by a 100 × oil immersion microscope objective with a high numerical aperture (NA) of 1.4.

### Materials

The performance of different photoresists in structure fabrication can be diverse due to their own unique chemical compositions and physical properties. In this work, photoresists called sol-gel organic-inorganic Zr-hybrid material [27] and E-shell 300 (Envisiontec) are applied respectively for the structuring. Zr-hybrid material is a high-viscosity zirconium-based sol-gel organic-inorganic hybrid polymer which is well known for its low shrinkage and high stability for 2PP fabrication. The preparation procedures and other optical properties of this photoresist can be found in ref [27]. E-shell 300 is a dimethacrylate-based liquid photoresist with a viscosity of 339.8 MP a·s. It can be used for 3D printing and fabrication of hearing aid and medical devices, as well as structures with high resolution, strength, stiffness, and chemical resistance.

## Results and Discussion

**Fig. 3:**
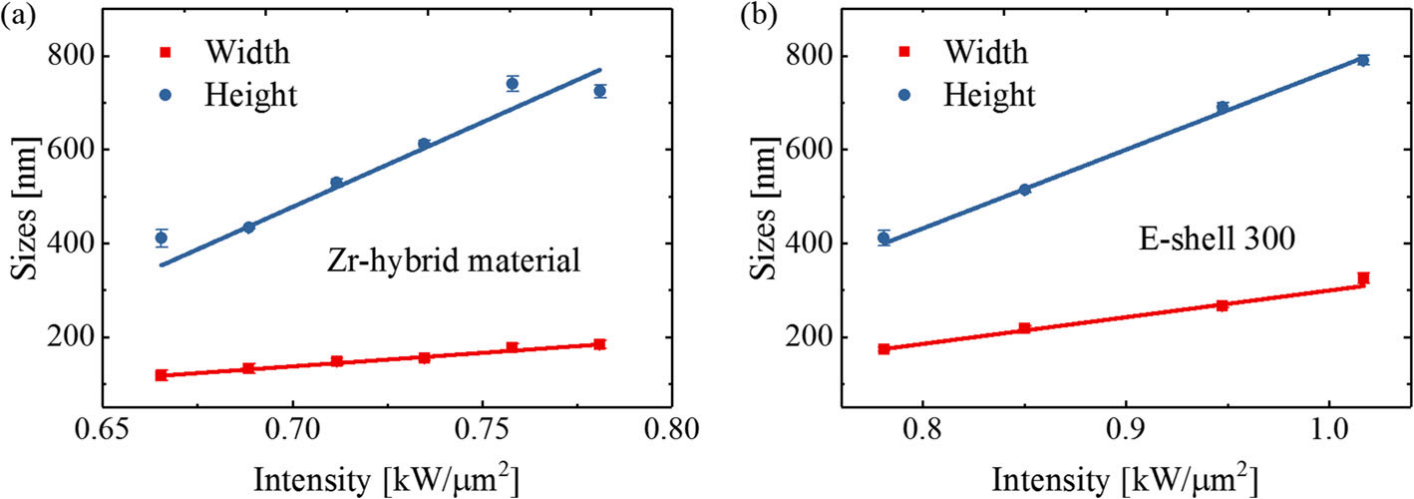
Line dimensions versus the laser intensity *I*. The speed used for the structuring is 7 *μ*m/s. The red and blue lines are linear fit results of voxel width and height, respectively. **a** The width and height of a single line made of Zr-hybrid material. **b** The width and height of a single line made of E-shell 300

The processing parameters play an important role in determining the feature sizes of structures. Among them, laser intensity is one parameter that is able to effectively influence the structure formation and can be controlled accurately and conveniently. This parameter can be obtained using the formula given in ref [28]

1 $$ {I=\frac{2 P T M^{2}}{\pi w_{0}^{2} f\tau}}  $$

where *P* represents the average laser power [4, 28], *T* the transmission coefficient of the objective/system (*T*=15*%* [4]), *M*^2^ the beam quality with *M*^2^=1.1, *f* the repetition rate, *τ* the pulse duration, and *w*_0_ the spot radius with $w_{0}=0.61 \frac {\lambda }{NA}$ (*w*_0_≈223.5 nm). In this formula, $\frac {P}{f}$ and $\frac {P}{f\tau }$ indicate the energy per pulse and average power per pulse, respectively. The intensity unit kW/ *μ*m^2^ is used instead of TW/cm^2^ (1 TW/cm ^2^=10 kW/ *μ*m^2^) for the purpose of straightforward displaying how much power is really focused in the spot area, which also has a range at microscale ($\pi w_{0}^{2} \approx 0.16$ *μ*m^2^). Here, an investigation about the effect of laser intensity on single line dimensions was carried out. Both Zr-hybrid material and E-shell 300 were applied for the study. The line width and height made of both materials with respect to the laser intensity *I* is shown respectively in Fig. 3a (Zr-hybrid material) and Fig. 3b (E-shell 300). A speed of 7 *μ*m/s was used for the fabrication. The laser intensity *I* is in the range 0.67–0.78 kW/ *μ*m^2^ (with a corresponding laser power range 1.44–1.69 mW) for Zr-hybrid material and 0.78–1.02 kW/ *μ*m^2^ (laser power range 1.69–2.20 mW) for E-shell 300. It can be seen that the feature sizes (both diameter and height) go up with the increase of laser intensity. In the case of Zr-hybrid material (Fig. 3a), with the laser intensity of approximately 0.67 kW/ *μ*m^2^, the lateral dimension of a voxel can be reduced to around 115 nm, which is below the diffraction limit (the diffraction limit $\frac {\lambda }{2NA}=185$ nm). It can also be calculated that the aspect ratio (height to width) is in the range 2.5–4. For E-shell 300 (Fig. 3b), a line width of 178 nm was realized when laser intensity was 0.78 kW/ *μ*m^2^. This feature dimension is below the diffraction limit (185 nm). Based on the above investigation, it can be concluded that the feature sizes are effectively influenced by the applied laser intensity. A smaller feature size can be realized by reducing the laser intensity.

### Influence of Different Materials on the Structure Formation by 2PP

**Fig. 4:**
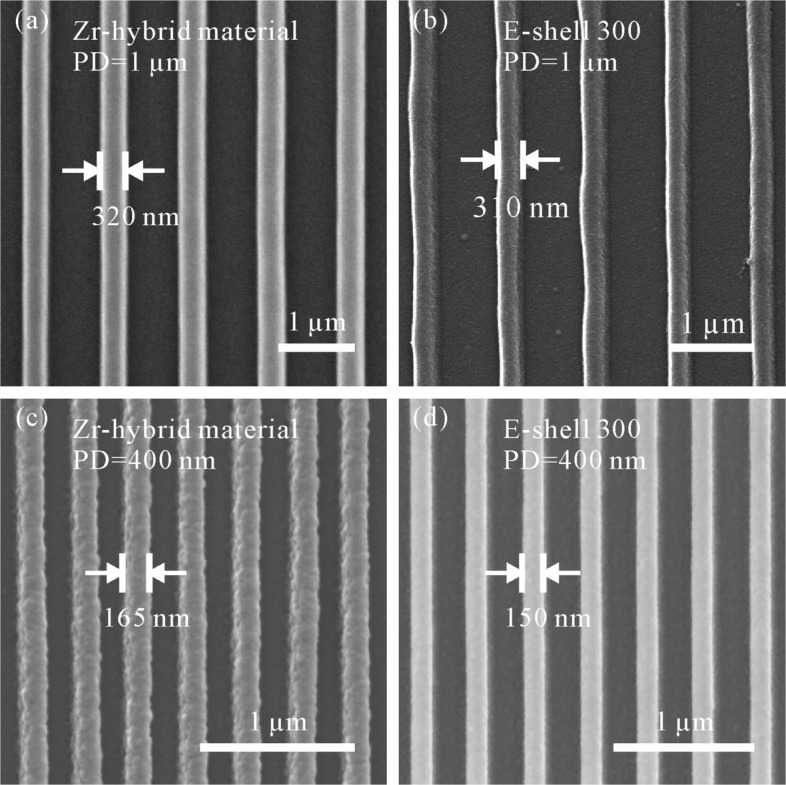
SEM images of grating lines fabricated with different materials. The speed for the fabrication is 7 *μ*m/s. **a** Material: Zr-hybrid material; *PD*=1 *μ*m; Laser intensity: 1.25 kW/ *μ*m^2^. **b** Material: E-shell 300; *PD*=1 *μ*m; Laser intensity: 1.02 kW/ *μ*m^2^. **c** Material: Zr-hybrid material; *PD*=400 nm; Laser intensity: 0.69 kW/ *μ*m^2^. **d** Material: E-shell 300; *PD*=400 nm; Laser intensity: 0.69 kW/ *μ*m^2^

For the investigation on the impact of materials on structure formation, various periodic grating lines were fabricated using the materials introduced in “Materials” section. A writing speed of 7 *μ*m/s was applied. Figure 4a and b are respectively the SEM images of periodic grating lines made of Zr-hybrid material and E-shell 300 with the periodic distance (*PD*, illustrated in Fig. 1) of 1 *μ*m. Laser intensity applied for the fabrication was 1.25 kW/ *μ*m^2^ (corresponding to laser power 2.7 mW) for Zr-hybrid material and 1.02 kW/ *μ*m^2^ (corresponding to laser power 2.2 mW) for E-shell 300. It can be seen that the grating lines made of both materials are smooth. Figure 4c and d indicate the SEM images of periodic grating lines made of Zr-hybrid material and E-shell 300 with *PD*=400 nm, respectively. With the decrease of periodic distance, laser intensity used for the fabrication is reduced as well in order to achieve high resolution and simultaneously avoid overpolymerization inside the space between adjacent features. In this investigation, laser intensity of 0.69 kW/ *μ*m^2^ was applied for the fabrication with both materials. With the reduced *PD*, the grating lines made of Zr-hybrid material are grainy (Fig. 4c), while that made of E-shell 300 have less roughness (Fig. 4d). The graininess of grating lines made of Zr-hybrid material might result from an unstable polymerization, which happens due to the proximity of reduced laser power to the polymerization threshold of the material. This comparison reveals that E-shell 300 is more suitable for the fabrication of structures with a nanoscale periodic distance. In addition, all of the structures observed by SEM are deposited with a 20-nm-thick gold layer.

### Investigation of Structure Formation with Respect to the Laser Focus Position

To place the nanostructures on the surface of the glass substrate, the laser beam has to be focused at the substrate/photoresist interface during the 2PP process. Thus, only part of the voxel is able to initiate the polymerization of photoresist. The other part of the voxel is in glass substrate to ensure the adhesion of structures. Since the voxel geometry is elliptical, a variation of its cross-section size exists along the major axis. In high-resolution micro- and nanofabrication, the variation of voxel cross-section size at the interface of substrate and photoresist is of much concern in affecting the structure formation as well as its feature size.

**Fig. 5:**
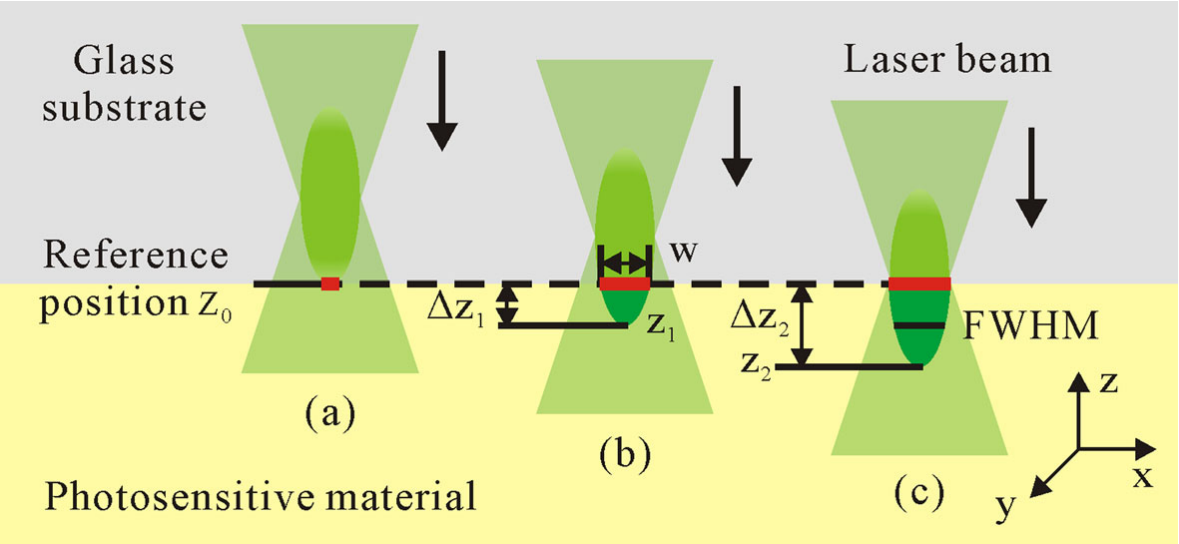
Illustration of the variation of laser focus position along *z* direction

Figure 5 is a schematic illustration of laser focus adjustment along *z* direction. The position at the interface between the photoresist and the substrate is defined as a reference focus position *z*_0_ (Fig. 5a). Since photoresist droplet is on the bottom side of the glass substrate, laser focus spot moves down from the reference position *z*_0_ into the photoresist. The distance between the current laser focus position *z* and the reference position *z*_0_ is represented by Δ*z*=∣*z*−*z*_0_∣. The region indicated with dark green color in Fig. 5b and c represents the laser focus region inside the photoresist, which enables the polymerization with light intensity above the polymerization threshold. Different feature sizes can be realized by placing laser focus at different *z* positions. Feature size *w* is characterized by the average full width half maximum (FWHM, Fig. 5c) of the features that are fabricated at the same *z* position in one array.

**Fig. 6:**
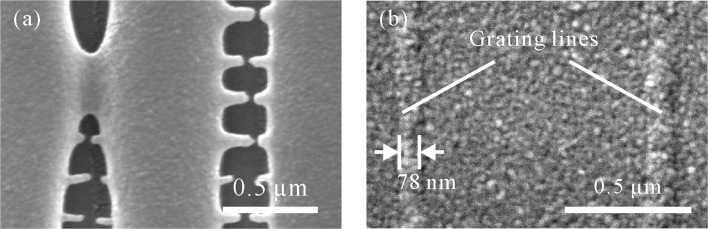
The influence of laser focus positions on structure formation. Material: E-shell 300. **a** Vertical grating lines fabricated with laser focus more inside the photoresist. The laser intensity for fabrication *I*=0.71 kW/ *μ*m^2^ (corresponding to laser power 1.55 mW), the relative laser focus distance *Δz*=500 nm. Extra polymerization between the features is generated, and the adjacent features are connected. **b** Vertical grating lines fabricated with laser focus more inside the substrate. The laser intensity for fabrication *I*=0.65 kW/ *μ*m^2^ (corresponding to laser power 1.4 mW), the relative laser focus position *Δz*=0 nm

Periodic grating lines fabricated with different laser focus positions were obtained as presented in Fig. 6. The periodic distance (*PD*) between grating lines is 1 *μ*m. With this close *PD*, the adjacent features begin to connect to each other through extra polymerization in the gap region when laser is focused with *Δz*=500 nm (Fig. 6a). The clusters out of the grating lines result from additional polymerization. During the 2PP process, free radicals are generated through the laser-induced bond cleavage in the photoinitiator molecules. Those radicals are accumulated in the small gaps between the adjacent features, which results in the increase of the radical concentration. This high radical concentration can exceed the polymerization threshold and thus lead to undesired polymerization. Moreover, an unstable adhesion of polymerized structures to the substrate can also be resulted. In this case, the structures can be easily washed away during the development process. When the focus of laser beam is more inside the substrate, less photoresist is polymerized. As presented in Fig. 6b, grating lines with the width of 78 nm was achieved in this case. However, weak visibility of the structure can also be seen. Therefore, it is of great importance to have a proper laser focus position during the polymerization process not only for a higher resolution but also for a better adhesion of structure to the substrate.

**Fig. 7:**
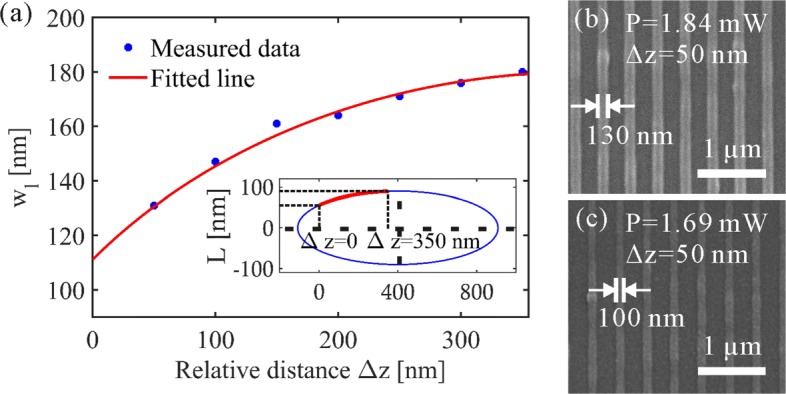
Grating lines fabricated at the *x*−*y* plane with respect to different relative laser focus distance Δ*z*. Material: E-shell 300. A writing speed of 7 *μ*m/s was applied. **a** Measured line width and fitted curve with respect to different Δ*z*. The figure in the lower right corner is a reconstruction of the ellipse corresponding to the fitted line. **b** Grating lines fabricated with the laser intensity of *I*=0.85 kW/ *μ*m^2^ (with the laser power *P*=1.84 mW). The relative laser focus distance is Δ*z*=50 nm. **c** Grating lines fabricated with the laser intensity of *I*=0.78 kW/ *μ*m^2^ (with the laser power *P*=1.69 mW). The relative laser focus distance is Δ*z*=50 nm

As to the influence of laser focus position on the feature sizes, an investigation of its effect on the grating lines that are fabricated in the *x*−*y* plane was conducted. By increasing the relative distance Δ*z*, grating lines fabricated under different laser focus positions were obtained. The measured width of grating lines *w*_*l*_ depending on the relative laser focus positions is plotted as the dots presented in Fig. 7a. Laser intensity used for the fabrication is 0.85 kW/ *μ*m^2^ (corresponding to laser power 1.84 mW). The red curve indicates an elliptical fit result in which the major axis is consistent with *z* axis. The corresponding ellipse was reconstructed (see the lower right corner of Fig. 7a) using the elliptical formula $\left (\frac {x}{a}\right)^{2}+\left (\frac {400-y}{b}\right)^{2}=1$, where (400,0) is the center of the ellipse, *b*=90 is the semi-minor axis, *a*=5.65*b* is the semi-major axis, *x* represents the relative distance Δ*z* along the major axis, and *y* represents half of the focus size *L* which is along the minor axis. The result reveals that the line width follows with the laser focus cross-section size which changes along the major axis of the voxel elliptical geometry. When the relative position Δ*z*=50 nm, grating lines with a feature size of *w*_*l*_=130 nm were realized (Fig. 7b). Additionally, by reducing the laser intensity, grating lines with *w*_*l*_=100 nm were obtained at the same laser focus position as presented in Fig. 7c.

**Fig. 8:**
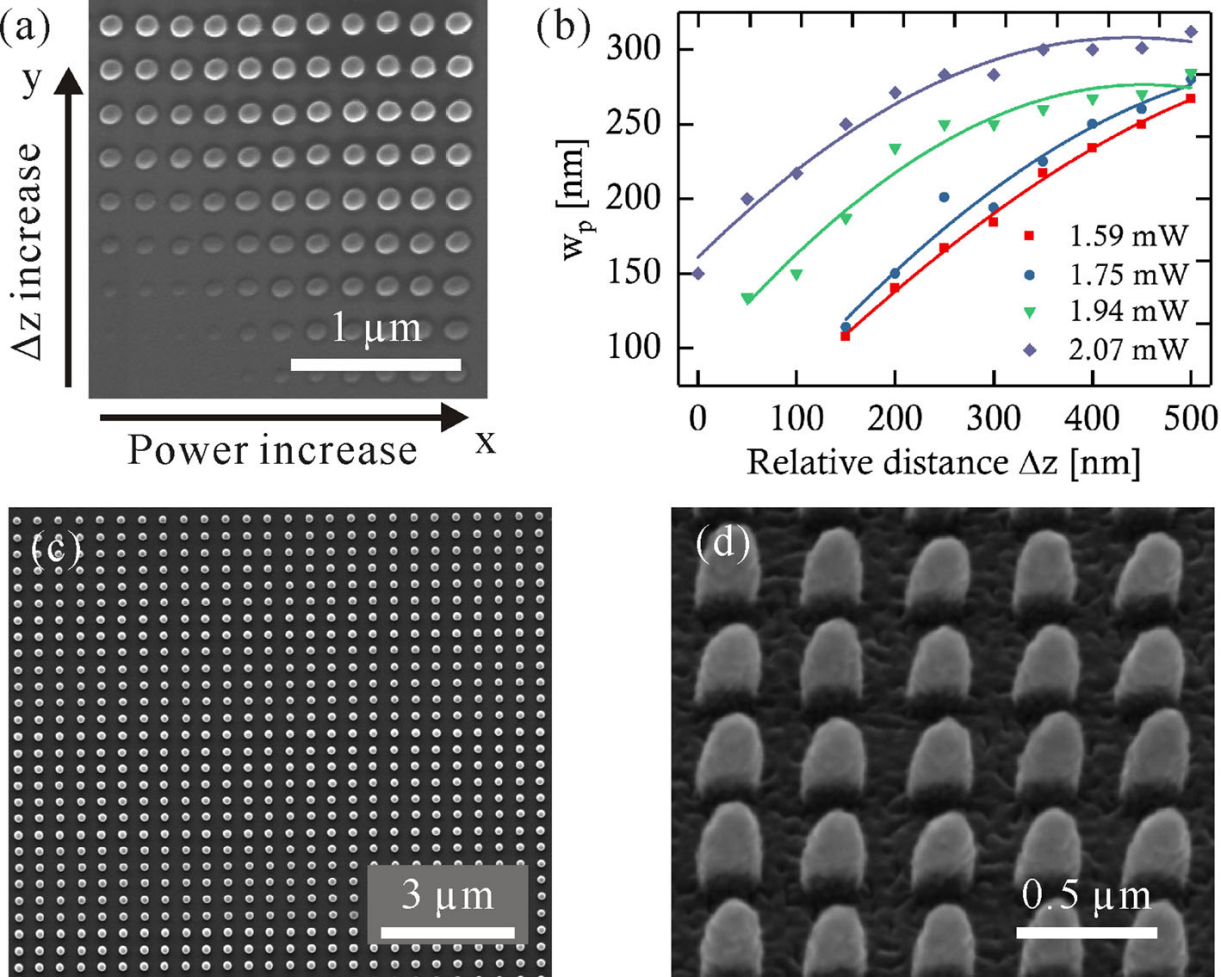
Pillar arrays fabricated with different laser intensity and laser focus relative distance Δ*z*. Material: E-shell 300. **a** SEM image of pillars fabricated with different laser intensity and relative laser focus positions. **b** Measured pillars diameter *w*_*p*_ with respect to the laser intensity *I* and the relative distance Δ*z*. Laser intensity is respectively 0.74 kW/ *μ*m^2^, 0.81 kW/ *μ*m^2^, 0.90 kW/ *μ*m^2^, and 0.96 kW/ *μ*m^2^ with the correspondence of laser power 1.59 mW, 1.75 mW, 1.94 mW, and 2.07 mW. **c** Top view of the pillar array. **d** SEM image of the pillar array viewed with 45 ^∘^

The influence of laser focus position on the feature sizes of pillars was also investigated. The pillars are realized by moving the focal spot orthogonally to the substrate plane, which is in the plane of the major axis of voxel (*x*−*z* or *y*−*z* plane). A single pillar was fabricated by moving the laser beam along *z* direction with a distance of 1 *μ*m. Figure 8a is the SEM image of pillars manufactured with different laser intensity and relative distances Δ*z*. The distance between the centers of adjacent pillars is 400 nm along *x* direction and 500 nm along *y* direction. Laser intensity was increased from the left to the right with a step of approximately 0.23 kW/ *μ*m^2^ (corresponding to laser power 0.5 mW). The relative distance between the laser focus position *z* and the reference position *z*_0_ was increased from the bottom to the top along the vertical direction. Figure 8b shows measured pillar diameters *w*_*p*_ regarding the laser intensity and the relative distance Δ*z*. The diameter of a pillar *w*_*p*_ is obtained by measuring its FWHM. The laser intensity is in the range 0.74–0.96 kW/ *μ*m^2^. It can be observed that *w*_*p*_ is reduced with the decrease of both Δ*z* and the laser intensity. When Δ*z*=150 nm, a pillar with the diameter of *w*_*p*_≈110 nm was achieved with a relatively large laser intensity range (0.74–0.81 kW/ *μ*m^2^). And there is also a relatively stable window for the pillar sizes when an array of pillars is fabricated as presented in Fig. 8c–d, which is the SEM images of a pillar array fabricated with the laser intensity of *I*=0.74 kW/ *μ*m^2^ and a relative distance of Δ*z*=300 nm. The aspect ratio of the pillar is around 2. It indicates that the reproducibility of pillar performs very well.

### Fabrication of Periodic Structures with the Feature Sizes and Gap Size Below the Diffraction Limit

**Fig. 9:**
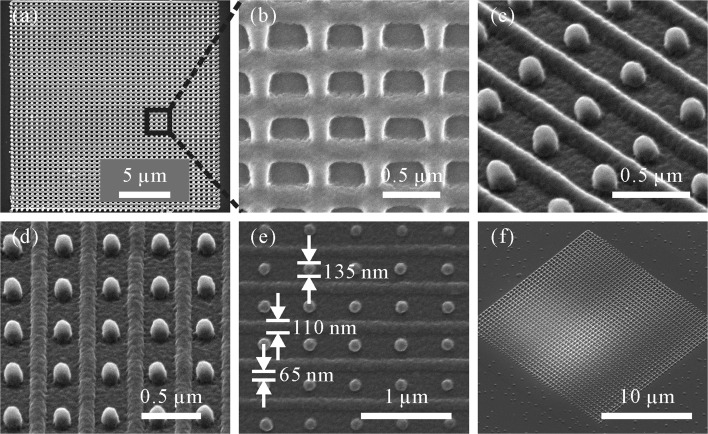
SEM images of 2PP fabricated periodic structure with *PD*=200 nm. Material: E-shell 300. Intensity used for the fabrication of grating lines: *I*=0.83 kW/ *μ*m^2^; pillars: *I*=0.6 kW/ *μ*m^2^. The relative laser focus distance for fabrication of grating lines and pillars is 300 nm. **a**–**b** Periodic structures fabricated with laser focus position setting inside the photoresist. **c**–**d** SEM images of periodic structures with proper laser focus position. **e** Top view of the structure fabricated with proper laser focus position. **f** SEM image of the whole array

Based on the respective investigations on the feature sizes of periodic grating lines (fabricated at *x*−*y* plane) and pillars, the proposed high-resolution periodic structure composed of grating lines and pillars was fabricated. Its size is 20×20 *μ*m with a periodic distance of 200 nm between the center of the grating line and the pillar. In this work, the strategy of achieving high-resolution structures with a periodic distance of 200 nm by separately fabricating grating lines and pillars is put forward. In this case, the periodic distance *PD* between adjacent grating lines and adjacent pillars is 400 nm. During the polymerization process, a larger gap region exists between the features when grating lines and pillars are fabricated separately. This temporarily broaden gap region enables to reduce the accumulation of radicals, which might lead to the undesired polymerization in the gap region. It has to be noted that the laser focus position also has to be adjusted during the fabrication process. Structures fabricated with improper laser focus position are presented in Fig. 9a and b. It can be seen that the lines and pillars are connected when the laser focus is too much inside the photoresist. Figure 9c–f are the SEM images of structures with well-positioned laser focus [23]. By placing the laser focus position properly and utilizing the fabrication strategy provided above, a structure with dimensions below the diffraction limit (a line width of 110 nm, a pillar diameter of 135 nm and a gap size of 65 nm) was realized as shown in Fig. 9e.

## Conclusion

In conclusion, we compared the influence of different photoresists and processing parameters on the structure formation and presented the way of improving the spatial resolution and reducing the gap size between adjacent features by controlling the laser focus position along *z* direction. E-shell 300 was experimentally proved to be a more suitable material for the fabrication of structures with a spatial resolution less than 200 nm. We also succeeded to achieve a periodic structure with the gap size of 65 nm and the feature size of 110 nm. The sizes are far below the Abbe diffraction limit. The further investigation on the optical performance (e.g., signal enhancement of optical images) of this high-resolution structure will be attractive.

## Abbreviations

2PA: Two-photon absorption
2PP: Two-photon polymerization
FWHM: Full width half maximum
IFTS: Interferometric Fourier transform scatterometry
NA: Numerical aperture
PD: Periodic distance
SEM: Scanning electron microscope

## References

[1] Seidel A, Ohrt C, Passinger S, Reinhardt C, Kiyan R, Chichkov BN (2009). Nanoimprinting of dielectric loaded surface-plasmon-polariton waveguides using masters fabricated by 2-photon polymerization technique. JOSA B.

[2] Malinauskas M, Gilbergs H, žukauskas A, Purlys V, Paipulas D, Gadonas R (2010). A femtosecond laser-induced two-photon photopolymerization technique for structuring microlenses. J Opt.

[3] Sun HB, Matsuo S, Misawa H (1999). Three-dimensional photonic crystal structures achieved with two-photon-absorption photopolymerization of resin. Appl Phys Lett.

[4] Serbin J, Egbert A, Ostendorf A, Chichkov B, Houbertz R, Domann G, Schulz J, Cronauer C, Fröhlich L, Popall M (2003). Femtosecond laser-induced two-photon polymerization of inorganic–organic hybrid materials for applications in photonics. Opt Lett.

[5] Gittard SD, Nguyen A, Obata K, Koroleva A, Narayan RJ, Chichkov BN (2011). Fabrication of microscale medical devices by two-photon polymerization with multiple foci via a spatial light modulator. Biomed Opt Express.

[6] Raimondi MT, Eaton SM, Nava MM, Laganà M, Cerullo G, Osellame R (2012). Two-photon laser polymerization: from fundamentals to biomedical application in tissue engineering and regenerative medicine. J Appl Biomater Biomech.

[7] Adams W, Sadatgol M, Güney DÖ (2016). Review of near-field optics and superlenses for sub-diffraction-limited nano-imaging. AIP Adv.

[8] Maznev A, Wright O (2017). Upholding the diffraction limit in the focusing of light and sound. Wave Motion.

[9] Heinzelmann H, Pohl D (1994). Scanning near-field optical microscopy. Appl Phys A.

[10] Bharadwaj P, Deutsch B, Novotny L (2009). Optical antennas. Adv Opt Photon.

[11] Ropers C, Neacsu C, Elsaesser T, Albrecht M, Raschke M, Lienau C (2007). Grating-coupling of surface plasmons onto metallic tips: a nanoconfined light source. Nano Lett.

[12] Ostendorf A, Chichkov BN (2006). Two-photon polymerization: a new approach to micromachining. Photonics Spectra.

[13] Xing JF, Dong XZ, Chen WQ, Duan XM, Takeyasu N, Tanaka T, Kawata S (2007). Improving spatial resolution of two-photon microfabrication by using photoinitiator with high initiating efficiency. Appl Phys Lett.

[14] Li L, Gattass RR, Gershgoren E, Hwang H, Fourkas JT (2009). Achieving *λ*/20 resolution by one-color initiation and deactivation of polymerization. Science.

[15] Emons M, Obata K, Binhammer T, Ovsianikov A, Chichkov BN, Morgner U (2012). Two-photon polymerization technique with sub-50 nm resolution by sub-10 fs laser pulses. Opt Mater Express.

[16] Haske W, Chen VW, Hales JM, Dong W, Barlow S, Marder SR, Perry JW (2007). 65 nm feature sizes using visible wavelength 3-D multiphoton lithography. Opt Express.

[17] Burmeister F, Steenhusen S, Houbertz R, Zeitner UD, Nolte S, Tünnermann A (2012). Materials and technologies for fabrication of three-dimensional microstructures with sub-100 nm feature sizes by two-photon polymerization. J Laser Applic.

[18] Gan Z, Cao Y, Evans RA, Gu M (2013). Three-dimensional deep sub-diffraction optical beam lithography with 9 nm feature size. Nat Commun.

[19] Fischer J, Wegener M (2011). Three-dimensional direct laser writing inspired by stimulated-emission-depletion microscopy. Opt Mater Express.

[20] Sakellari I, Kabouraki E, Gray D, Purlys V, Fotakis C, Pikulin A, Bityurin N, Vamvakaki M, Farsari M (2012). Diffusion-assisted high-resolution direct femtosecond laser writing. Acs Nano.

[21] Gailevičius D, Padolskytė V, Mikoliūnaitė L, Šakirzanovas S, Juodkazis S, Malinauskas M (2019) Additive-manufacturing of 3D glass-ceramics down to nanoscale resolution. Nanoscale Horiz.

[22] Paz VF, Peterhänsel S, Frenner K, Osten W, Ovsianikov A, Obata K, Chichkov B (2011) Depth sensitive fourier-scatterometry for the characterization of sub-100 nm periodic structures In: Modeling Aspects in Optical Metrology III, vol. 8083. International Society for Optics and Photonics, 80830.

[23] Reinhardt C, Paz VF, Zheng L, Kurselis K, Birr T, Zywietz U, Chichkov B, Frenner K, Osten W (2015) 4 design and fabrication of near-to far-field transformers by sub-100 nm two-photon polymerization In: Optically Induced Nanostructures: Biomedical and Technical Applications, 73.. Walter de Gruyter GmbH & Co KG. 26491776

[24] Rekštytė S, Jonavičius T, Gailevičius D, Malinauskas M, Mizeikis V, Gamaly EG, Juodkazis S (2016). Nanoscale precision of 3D polymerization via polarization control. Adv Opt Mater.

[25] Farsari M, Chichkov BN (2009). Materials processing: two-photon fabrication. Nat Photon.

[26] Zheng L, Kurselis K, Reinhardt C, Kiyan R, Evlyukhin A, Hinze U, Chichkov B (2017) Fabrication of sub-150 nm structures by two-photon polymerization for plasmon excitation In: Progress In Electromagnetics Research Symposium-Spring (PIERS), 2017, 3402–3405.. IEEE.

[27] Ovsianikov A, Viertl J, Chichkov B, Oubaha M, MacCraith B, Sakellari I, Giakoumaki A, Gray D, Vamvakaki M, Farsari M (2008). Ultra-low shrinkage hybrid photosensitive material for two-photon polymerization microfabrication. Acs Nano.

[28] Jonušauskas L, Juodkazis S, Malinauskas M (2018). Optical 3D printing: bridging the gaps in the mesoscale. J Opt.

